# The Anti-mycobacterial Activity of a Diterpenoid-Like Molecule Operates Through Nitrogen and Amino Acid Starvation

**DOI:** 10.3389/fmicb.2019.01444

**Published:** 2019-06-25

**Authors:** Alessandra Crusco, Rafael Baptista, Sumana Bhowmick, Manfred Beckmann, Luis A. J. Mur, Andrew D. Westwell, Karl F. Hoffmann

**Affiliations:** ^1^Institute of Biological, Environmental and Rural Sciences, Aberystwyth University, Aberystwyth, United Kingdom; ^2^School of Pharmacy and Pharmaceutical Sciences, Cardiff University, Cardiff, United Kingdom

**Keywords:** terpenoids, diterpenoids, mycobacteria, tuberculosis, *Mycobacterium smegmatis*, untargeted metabolomics

## Abstract

A library of 14 minimally cytotoxic diterpenoid-like compounds (CC_50_ > 70 μM on HepG2 human liver cells) was screened against *Mycobacterium smegmatis*, *Staphylococcus aureus*, and *Escherichia coli* to determine antimicrobial activity. Some compounds with a phenethyl alcohol (PE) core substituted with a β-cyclocitral derivative demonstrated anti-mycobacterial activity, with the most active being compound **1** (MIC = 23.4 mg/L, IC_50_ = 0.6 mg/L). Lower activity was exhibited against *S. aureus*, while no activity was displayed against *E. coli.* Low cytotoxicity was re-confirmed on HepG2 cells and additionally on RAW 264.7 murine macrophages (SI for both cell lines > 38). The sub-lethal (IC_50_ at 6 h) effect of compound **1** on *M. smegmatis* was examined through untargeted metabolomics and compared to untreated bacteria and bacteria treated with sub-lethal (IC_50_ at 6 h) concentrations of the antituberculosis drugs ethambutol, isoniazid, kanamycin, and streptomycin. The study revealed that compound **1** acts differently from the reference antibiotics and that it significantly affects amino acid, nitrogen, nucleotides and folate-dependent one-carbon metabolism of *M. smegmatis*, giving some insights about the mode of action of this molecule. A future medicinal chemistry optimization of this new anti-mycobacterial core could lead to more potent molecules.

## Introduction

Tuberculosis (TB), a highly infectious disease caused by the *Mycobacterium tuberculosis* (MTB), is included amongst the top 10 causes of human mortality according to the World Health Organization (World Health Organization [WHO], 2018). The first anti-TB agent to be discovered was streptomycin, but MTB resistance to this drug quickly evolved (Lienhardt et al., 2012). Current treatment for TB includes a 6-month combination therapy including the first-line drugs rifampicin, isoniazid, pyrazinamide and ethambutol (Lienhardt et al., 2012). However, multidrug-resistant TB (MDR-TB) has also developed, requiring affected patients to subsequently undergo 20 months of chemotherapy with more toxic second-line drugs; these regimes are associated with lower treatment success as well as emergence of additional resistance to certain second line drugs (kanamycin and fluoroquinolones) and the evolution of extensive drug resistant (XDR) TB (Mwanzia et al., 2015). As patients affected by MRD-TB and XDR-TB have low chances of survival, the discovery of new anti-TB drugs is an urgent priority.

In this study, a library of minimally cytotoxic diterpenoid-like compounds (CC_50_ > 70 μM on HepG2 human liver cells), previously synthesized and tested for anthelmintic activity (Crusco et al., 2018), was screened against *Mycobacterium smegmatis. M. smegmatis* is a non-pathogenic and fast-growing *Mycobacterium* that demonstrates drug-response characteristics comparable to the highly infectious MDR-TB and, for these characteristics, represents a suitable model for anti-MTB drug discovery (Chaturvedi et al., 2007). In particular, some compounds containing a phenethyl alcohol (PE) core substituted with a β-cyclocitral (cyclohexenyl) derivative showed anti-mycobacterial activity, with the most active being compound **1** (MIC = 23.4 mg/L, IC_50_ = 0.6 mg/L). Compounds were also screened against representative Gram positive (*Staphylococcus aureus*) and Gram negative (*Escherichia coli*) bacteria to ascertain generic antimicrobial properties. In contrast to *M. smegmatis*, low to no activity was found for *S. aureus* and *E. coli* suggesting some degree of diterpenoid selectivity. As PE is a known antimicrobial (Corre et al., 1990; Lucchini et al., 1990), the anti-mycobacterial activity of this compound was additionally tested. Here, the MIC was found to be > 125 mg/L suggesting that the β-cyclocitral derivative substituent and/or the 4-methoxy aromatic substituent are essential for the anti-mycobacterial activity.

Metabolomics-based investigations of compound **1**’s anti-mycobacterial activity indicated modulation of amino acid, nitrogen, nucleotides, and folate-dependent one-carbon metabolism within the bacteria that was not observed with other tested established antibiotics. In particular, a condition of nitrogen/amino acid starvation was identified, which could be at the base of compound **1**’s mode of action.

In conclusion, we found a selective anti-mycobacterial core that, to the best of our knowledge, has not been explored yet and whose optimization could bring to the development of more potent anti-mycobacterial compounds. The mechanism of action of this class of molecules has been investigated by untargeted metabolomics revealing a significant decrease in nitrogen-containing metabolites and a possible nitrogen/amino acid starvation effect. Further studies will be necessary to confirm the generated hypothesis and to give new insights on this family of molecules.

## Materials and Methods

### Chemicals

The diterpenoid library was synthesized as described previously (Crusco et al., 2018). Chiral compounds (**1**, **3**, **6**, **8**, **10**, **11**, **12**, **13**, and **14**) were synthesized and tested as racemic mixtures (Crusco et al., 2018). After initial screening (see paragraph below), compound **1** was resynthesized and retested to confirm the antimicrobial activity. Streptomycin sulfate, ethambutol dihydrochloride, isoniazid and phenetyl alcohol were obtained from Sigma. Kanamycin sulfate was obtained from Gibco. All compounds, the synthetic diterpenoids and the standard anti-TB drugs, were solubilized as stock solutions of 2.5 mg/mL in (1:1) methanol/water.

### Bacterial Growth, Minimum Inhibitory Concentration (MIC) Calculation, and IC_50_ Determination

All procedures were performed in a biosafety level 2 (BSL2) cabinet. *S. aureus* ATCC 29213 and *E. coli* ATCC 25922 were cultured in Luria-Bertani (LB) medium at 37°C with aeration at 200 rpm for 24 h, while *M. smegmatis* mc^2^155 was cultured in LB medium supplemented with 0.2% (v/v) glycerol and 0.05% (v/v) Tween 80 at 37°C with aeration at 200 rpm for 48 h. The stationary phase cultures were then used for minimum inhibitory concentration (MIC) determination using the broth microdilution method, in fresh LB medium, in a 96-well plate (European Committee for Antimicrobial Susceptibility Testing [EUCAST] of the European Society of Clinical Microbiology and Infectious Diseases [ESCMID], 2003). All compounds were tested in triplicate using an initial bacterial concentration of 5.0 × 10^5^ colony forming units (CFU)/mL at final concentrations of 250 mg/L and 125 mg/L (5% and 2.5% v/v methanol). Compounds with no visible growth at 125 mg/L were further evaluated with progressing dilutions. The MIC was determined as the lowest concentration of a compound at which no growth was visible after 24 h (*S. aureus* and *E. coli*) or after 48 h (*M. smegmatis*). For compound **1**, dilutions were repeated in three independent experiments where the optical density (OD_600_) was measured in a Hidex plate spectrophotometer and absorbance data were used for the calculation of an IC_50_ value. The IC_50_ value represents a compound concentration that inhibits 50% of bacterial growth (negative control as 100%) and was obtained from a dose response titration (250–0.09 mg/L). Dose response curves were obtained by non-linear regression, after log transformation of concentrations and data normalization using GraphPad Prism 7.02.

### Mammalian Cell Culture and MTT Assay

HepG2 human liver cancer cells were grown in BME culture media with the addition of 10% v/v Fetal Bovine Serum, 1% v/v MEM non-essential amino acid solution, 1% v/v 200 mM L-Glutamine, 1% v/v antibiotic/anti-mycotic. RAW 264.7 murine macrophage cells were grown in DMEM media with the addition of 10% v/v Fetal Bovine Serum, 1% v/v 200 mM L-Glutamine, 1% v/v antibiotic/anti-mycotic. When ∼80% confluency was reached, all cells were prepared for MTT viability assays as previously described (Nur-e-alam et al., 2017; Crusco et al., 2018). Briefly, 2.5 × 10^4^ cells per well were cultured in a black walled 96-well microtiter plate (Fisher Scientific, Loughborough, United Kingdom) and incubated for 24 h at 37°C in a humidified atmosphere with 5% CO_2_. Test compounds were then titrated from 100 to 3.13 μM (1.25 final % DMSO) and negative (DMSO; 1.25%) and positive (1% v/v Triton X-100) (Dayeh et al., 2004) controls included. After a 24 h incubation, the MTT assay was performed as described (Nur-e-alam et al., 2017; Crusco et al., 2018) and an IC_50_ value calculated from absorbance data as described in the previous paragraph.

### Metabolomics Sample Preparation and Metabolite Extraction

The procedure previously described by Baptista et al. (2018) was followed with minor modifications. Briefly, a total of 1.5 L of mycobacterial culture was incubated and grown in constant shaking at 200 rpm at 37°C. After 48 h, at OD_600_ = 0.6, thirty-six samples (6 biological replicates × 6 treatments; compound **1**, ethambutol, isoniazid, kanamycin, streptomycin and media only control) of 30 mL bacterial culture (10 mL × each time point; 0, 3, and 6 h) were dosed with compound **1** and antibiotics, at the concentration able to inhibit 50% of growth at OD_600_ = 0.6 after 6 h (31.25 mg/L for compound **1**, ethambutol and isoniazid, 7.3 mg/L for kanamycin, and streptomycin). At each time point, an aliquot of 10 mL culture was harvested from each sample, immersed in liquid nitrogen to quench bacterial metabolism and stored at -80°C. In preparation for the extraction, samples were thawed, centrifuged (10°C, 4500 rpm), washed with 0.85% NaCl and adjusted to an OD_600_ of 2. Extraction was performed by four freeze-thaw cycles and vortexing in 200 μL of a chloroform/methanol/water 1:3:1 solution. After a final centrifugation, 100 μL of solution were transferred in mass vials for flow infusion electrospray high-resolution mass spectrometry (FIE-HRMS) fingerprinting analysis.

### Metabolomics Analysis

FIE-HRMS was performed in the High Resolution Metabolomics Laboratory (HRML) at Aberystwyth University. A Q-Exactive Plus mass analyzer equipped with an UltiMate 3000 UHPLC system (Thermo Fisher Scientific) generated metabolite fingerprints in positive-negative polarity switching mode. Ion intensities were acquired between *m/z* 55 and 1200 in profiling mode at a resolution setting of 280,000 for 3.5 min. An auto sampler injected 20 μl extract into a flow of 100 μl^*^min^–1^ methanol:water (70:30, v/v). Electro spray ionization (ESI) source parameter settings were according to manufacturer’s recommendations. Mass spectra around the apex of the infusion maximum were combined into a single mean intensity matrix (runs x *m*/*z*) for each ionization mode using an in-house data aligning routine in Matlab (R2013b, The MathWorks). Data were log_10_-transformed before statistical analysis.

MetaboAnalyst 4.0. – Statistical analysis (Chong et al., 2018) was used to perform principal component (PCA). MetaboAnalyst 4.0 – MS peaks to pathway (Chong et al., 2018) was used to identify metabolites (tolerance = 3 ppm) and significant affected metabolic pathways (model organism = *S. aureus*). MetaboAnalyst pathway identification is based on *mummichog*, an algorithm able to predict biological activity directly from mass spectrometry data, avoiding the *a priori* identification of metabolites (Li et al., 2013). *Mummichog* plots all possible matches in the metabolic network and then looks for local enrichment, providing reproduction of true activity, as the false matches will distribute randomly (Li et al., 2013). Examples of key-metabolites in local enrichment were analyzed for significant difference (*t*-test) between control and treatment on Microsoft Excel and by GraphPad Prism 7.02.

## Results and Discussion

To investigate potential antimicrobial activities of the previously synthesized diterpenoids (Crusco et al., 2018), a total of 14 related compounds were analyzed for their physicochemical properties and screened against *S. aureus*, *E. coli* and *M. smegmatis* (Table 1). All the compounds showed a predicted good oral bioavailability and drug-likeness, according to property predictions performed by SwissADME (Daina et al., 2017) The most potent antimicrobial activity was found for compound **1** with a MIC of 23.4 mg/L on *M. smegmatis*, 62.5 mg/L on *S. aureus* and no activity on *E. coli* even at the highest concentration tested (250 mg/L). When compound **1** was compared to the other screened analogs, some trends in structure-activity relationship (SAR) were identified. Replacement of the alcoholic function (C-OH) with the corresponding oxidized keto form (C = O) considerably decreased the anti-mycobacterial activity (e.g., **1** vs. **2** and **3** vs. **4**), while the presence of the 4*-*methoxy substituent (-OCH_3_) on the phenyl ring was responsible for increased activity (e.g., **1** vs. **3**, **6**, **8**; **2** vs. **4**; **13** vs. **14**). As the molecules (**1** and **3**) sharing the alcoholic functions are structural analogs of PE, a known antimicrobial (Corre et al., 1990; Lucchini et al., 1990), this compound was also tested on *M. smegmatis.* However, PE was only active at the top concentration tested (250 mg/L), suggesting that the cyclo-hexenyl substituent and/or the 4-methoxy aromatic component of compounds **1** and **3** are the critical features associated with the anti-mycobacterial effects. The role of these substituents in the enhancement of the anti-mycobacterial activity is currently unknown, but may be linked to increased hydrophobic interactions of the molecule with a hypothetical target protein contained within *M. smegmatis*.

**TABLE 1:** Physicochemical properties and antibacterial activity of compound library.

**Compounds**	**Structures**	**RB**	**HBA**	**HBD**	**cLogP**	**MW**	**LRV**	***S. aureus***	***E. coli***	***M. smeg***
1	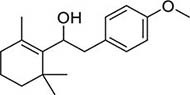	4	2	1	3.90	274.40	0	62.5	>250	23.4
2	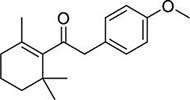	4	2	0	4.01	272.38	0	>250	>250	62.5
3	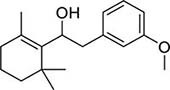	4	2	1	3.88	274.40	0	250	>250	31.25
4	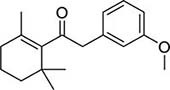	4	2	0	4.01	272.38	0	250	>250	125
5	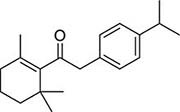	4	1	0	4.98	284.44	0	>250	>250	250
6	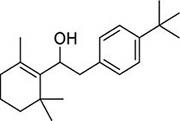	4	1	1	5.12	300.48	1	250	>250	250
7	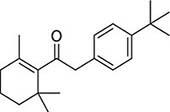	4	1	0	5.24	298.46	1	62.5	>250	250
8	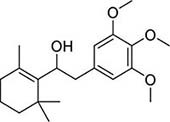	6	4	1	3.76	334.45	0	>250	>250	>250
9	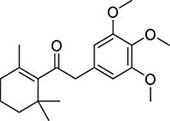	6	4	0	3.95	332.43	0	>250	>250	>250
10	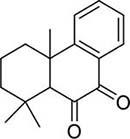	0	2	0	3.42	256.34	0	>250	>250	>250
11	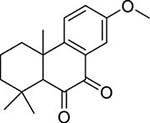	1	3	0	3.42	286.37	0	>250	>250	>250
12	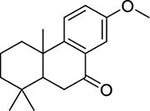	1	2	0	4.06	272.38	0	>250	>250	125
13	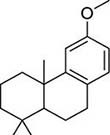	1	1	0	4.69	258.40	0	250	>250	62.5
14	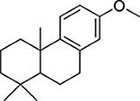	1	1	0	4.70	258.40	0	>250	>250	>250
PE	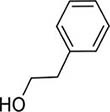	2	1	1	1.64	122.16	0	NA	NA	250

*Chemical structures and physicochemical properties of compound library calculated by SwissADME (Daina et al., 2017). RB, number of rotatable bonds; HBA, number of H-bond acceptors; HBD, number of H-bond donors; cLogP, consensus Log P; MW, molecular weight; and LRV, Lipinski’s rule violations. For predicted good oral bioavailability, values should be <10, <10, <5, ≤5, <500, and ≤1, respectively. Minimum inhibitory concentration (MIC; mg/L) of compound library against *Staphylococcus aureus*, *Escherichia coli* and *Mycobacterium smegmatis*. Values of >250 are considered to indicate no significant antimicrobial activity.*

As compound **1** demonstrated the best antimicrobial activity (*M. smegmatis* > *S. aureus* > *E. coli*), it was resynthesized (Crusco et al., 2018) and used in re-confirmatory experiments. Here, the anti-mycobacterial activity and the low HepG2 human liver cell cytoxicity were confirmed (Table 2). Furthermore, as MTB predominantly resides in alveolar macrophages (Dheda et al., 2016), compound **1**’s effect on RAW 264.7 murine macrophages was also determined (Table 2). Similar to findings in HepG2 cells, compound **1** exhibited low overt macrophage cytotoxicity (CC_50_ > 100 μM) and, consequently, high anti-mycobacterial selectivity (Table 2).

**TABLE 2:** Anti-mycobacterial and cytotoxic activity of compound **1**.

**Compound**	**MIC^a^**	**Viability of HepG2 cells at MIC^b^ (%)**	**Viability of RAW cells at MIC^b^ (%)**	**IC_50_^c^**	**CC_50_ on HepG2 cells^*a*^**	**CC_50_ on RAW cells^a^**	**Selectivity Index^d^**
1	23.4 [85.3]	104 ± 0.14	88 ± 0.29	0.6 (0.2–1.3)[2.2 (0.8–4.8)]	>27.4 [>100]	>27.4 [>100]	>38.5

*^*a*^Values expressed as mg/L [μM] and confirmed in triplicate. ^*b*^Values expressed as average percentage ± SD of three replicates. ^*c*^IC_50_ determined from three independent experiments and expressed as mg/L [μM] with 95% confidence interval. ^*d*^Calculated from IC_50_ on *M. smegmatis* when compared to the CC_50_ on mammalian cells.*

**FIGURE 1:**
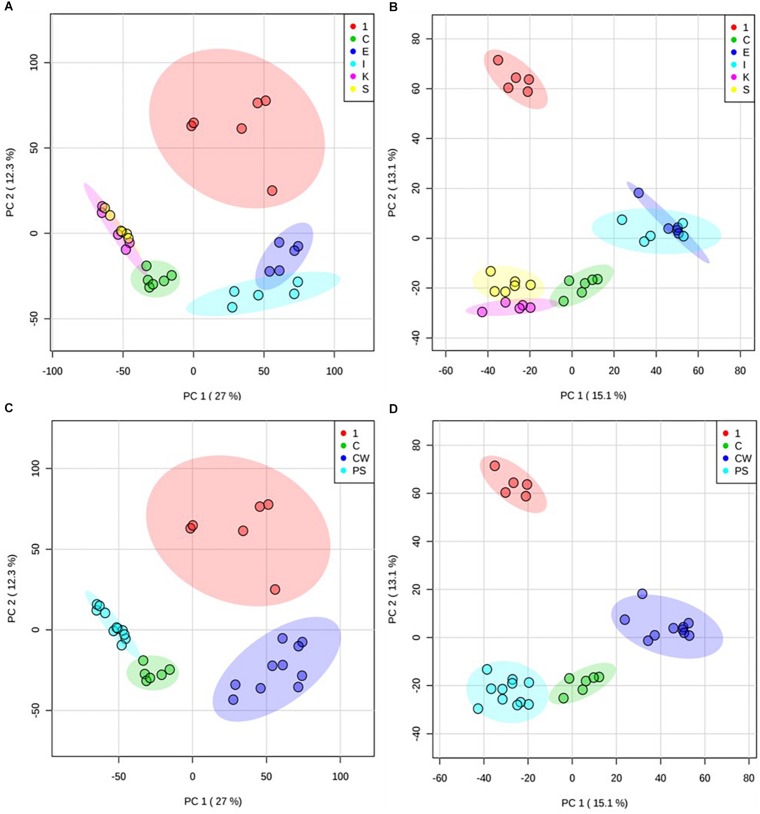
Principal component analysis (PCA) of treated *M. smegmatis* metabolome. PCA score plots (*n* = 6 and 95% confidence interval illustrated, clear outliers removed) of normalized *m/z* intensities of metabolites extracted from *M. smegmatis* treated with compound **1** (1) compared to control bacteria (C) and to bacteria treated with ethambutol (E), isoniazid (I), kanamycin (K), and streptomycin (S) for 6 h. Plots indicate metabolome differences between treatment groups based on metabolite features detected by FIE-HRMS in **(A)** positive and **(B)** negative ionization mode. Antibiotics with similar mechanism of action were grouped into those with activity on cell wall (ethambutol and isoniazid, CW) and on protein synthesis (kanamycin and streptomycin, PS) for both **(C)** positive and **(D)** negative mode. Compound **1** did not show any overlap with these antibiotics.

The activity of compound **1** was further investigated by examining metabolomic changes following its addition to *M. smegmatis* cultures (at its IC_50_ value at 6 h, 31.25 mg/L) for 6 h using methodologies previously described by Baptista et al. (2018) and further assessed in MetaboAnalyst 4.0 (Chong et al., 2018). Extracted metabolites derived from treated vs control *M. smegmatis* were profiled by using FIE-HRMS, a cutting edge high-throughput method used toward mode of action studies within bacteria. Unsupervised principal component analysis (PCA) of the identified *m/z* features indicated that the metabolome of bacteria treated with compound **1** was quite distinct from the metabolome of bacteria treated with ethambutol, isoniazid, kanamycin, and streptomycin (each also at IC_50_ values) as well as untreated bacteria (Figures 1A,B). Treatment of *M. smegmatis* with antibiotics having similar mechanisms of action (cell wall disruption by inhibition of mycolic acid synthesis for ethambutol and isoniazid (Chakraborty and Rhee, 2015); protein synthesis inhibition through binding to the 30 s subunit of ribosomes for streptomycin and kanamycin (Poulikakos and Falagas, 2013) showed overlapping metabolomics signatures. Accordingly, these signatures grouped further into two clusters, and sample profiles derived from those antibiotics acting on *M. smegmatis* cell wall components clustered away from those affecting protein synthesis (Figures 1C,D). Bacteria treated with compound **1** did not show any overlapping metabolomics signature with either the control bacteria or four antibiotic-treated bacteria, suggesting a different mechanism of action.

To identify the metabolites responsible for separating control- and compound **1**- treated *M. smegmatis* samples in PCA (at 6 h), the metabolome data of these two samples was analyzed using MetaboAnalyst 4.0 (Chong et al., 2018). A total of 1902 *m/z* features were differentially found between the two samples in the positive ionization mode, whereas 1456 *m/z* HRMS elements were differently present amongst the two samples in the negative ionization mode (*p* < 0.001). Examination on these signatures by MetaboAnalyst 4.0 – MS peaks to pathway revealed that amino acid-, nitrogen-, nucleotide- and folate-dependent one-carbon metabolism are the most significantly affected pathways in *M. smegmatis* samples treated with compound **1** (Table 3 for significant different pathways, Supplementary Information for all identified metabolites and pathways, Supplementary Tables S1–S4).

**TABLE 3:** Significantly affected pathways in *M. smegmatis* after 6 h treatment with compound **1**.

	**Pathway total**	**Hits total**	**Hits sig (*p* < 0.05)**	**Hits sig (*p* < 0.001)**	**EASE**	**FET**	**Gamma**
Aminoacyl-tRNA biosynthesis^a^	66	21	20	17	0.00188	0.00039	0.00127
Glycine, serine, and threonine metabolism^a^	26	16	15	13	0.00919	0.00198	0.00132
One carbon pool by folate^a^	7	6	6	6	0.05592	0.00627	0.00162
Valine, leucine, and isoleucine biosynthesis^a^	26	9	8	7	0.13526	0.03808	0.00233
Nitrogen metabolism^a^	14	9	9	7	0.13526	0.03808	0.00233
Valine, leucine, and isoleucine degradation^a^	25	11	9	8	0.13916	0.04582	0.00237
Arginine and proline metabolism^a^	30	17	16	11	0.13932	0.05897	0.00237
Alanine, aspartate, and glutamate metabolism^a^	18	13	12	9	0.14059	0.05159	0.00239
Cyanoamino acid metabolism^a^	8	4	4	4	0.21485	0.03422	0.00336
Valine, leucine, and isoleucine biosynthesis^b^	26	9	9	8	0.02494	0.00383	0.00234
Pyrimidine metabolism^b^	33	27	25	16	0.07320	0.03358	0.00286
Purine metabolism^b^	53	33	29	20	0.11988	0.06469	0.00347

*^*a*^From metabolites identified in positive ionization mode. ^*b*^From metabolites identified in negative ionization mode. Pathways identifications were performed by using MetaboAnalyst 4.0 (Chong et al., 2018) as described in the Section “Materials and Methods,” after analysis and identification (tolerance = 3 ppm) of *m/z* features obtained by FIE-HRMS. The table includes ranked enriched pathways, total number of hits, significant hits (*p* < 0.05 and *p* < 0.001), their EASE (Expression Analysis Systematic Explorer) score (Hosack et al., 2003) their raw *p*-values (Fisher’s Exact Test, FET) and the *p*-values using a Gamma distribution (all scores calculated based on the hits at *p* < 0.001).*

**FIGURE 2:**
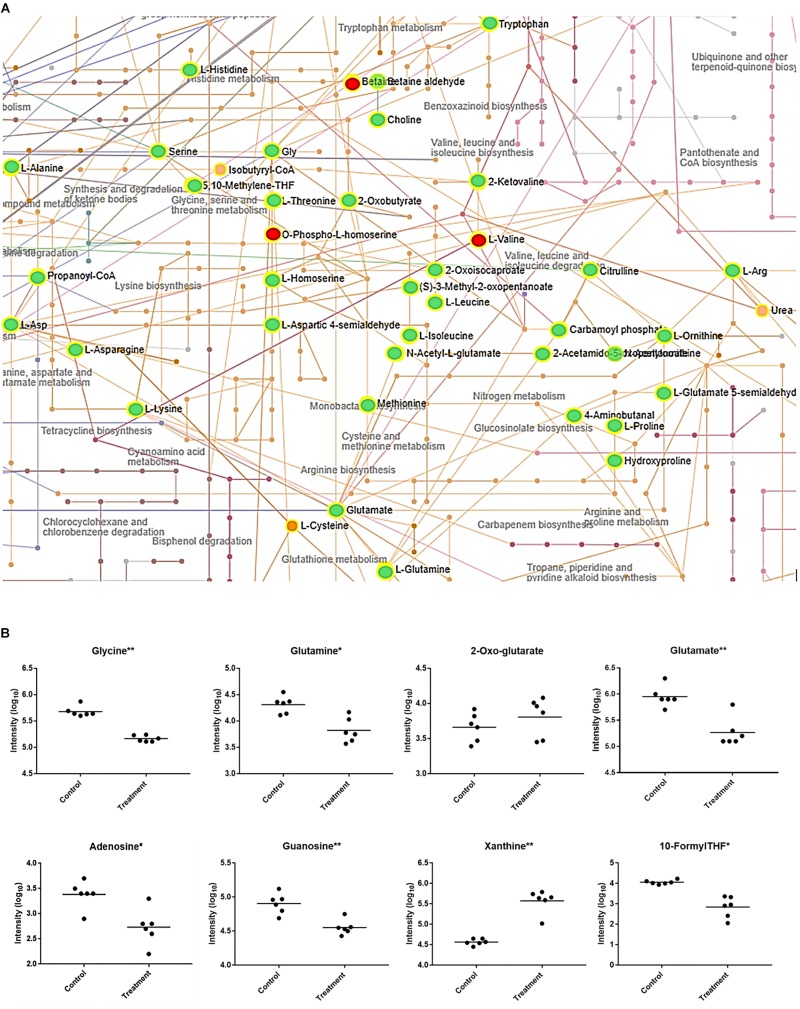
*Mycobacterium smegmatis* metabolic networks affected by compound **1**. **(A)** Compound **1** significantly affects amino acid and nitrogen metabolisms. All the significantly more abundant metabolites are colored in red, while the less abundant metabolites in green (when compared to the control *M. smegmatis* samples). Identified hits with no significant changes are in orange. Corresponding names for each metabolite and pathway are also annotated. For pyrimidine, purine and folate-dependent one-carbon metabolic networks see Supplementary Figure S2. **(B)** Some key metabolites with significantly (^*^p < 0.01, ^∗∗^*p* < 0.001) different concentrations between control and treatment are indicated.

Upon further interrogation of the statistically significant metabolites (Figure 2), most amino acids identified were less abundant in compound **1** treated *M. smegmatis* samples when compared to the controls, as well as metabolites belonging to nitrogen metabolism and the urea cycle (carbamoyl phosphate, citrulline, ornithine, glutamate-5-semialdehyde) (Figures 2A,B). This trend was not observed in the reference antibiotics treatments (Supplementary Figure S1). Significantly affected pathways also included pyrimidine metabolism and, on the limit of significance (*p* = 0.06), purine metabolism. In particular, most nucleosides (adenosine, guanosine, cytidine, uridine, thymidine and their mono and di-phosphate forms) were significantly less abundant, while some nucleoside metabolic products (xanthine, xanthosine monophosphate, adenine, phosphoribosyl pyrophosphate) were found to be more abundant in compound **1** treated *M. smegmatis* samples compared to controls (Figure 2B and Supplementary Figure S2A).

Collectively, the metabolomics data could suggest that compound **1**, and its related analogs, induces nitrogen and/or amino acid starvation. This would also impact on purine and pyrimidine synthesis through their dependence on glutamine and glutamate. Therefore, the lower concentrations of nucleosides and the increased concentration of a nucleoside precursor (phosphoribosyl pyrophosphate) could suggest a downregulation of nucleoside biosynthesis. Equally, this data could suggest that increased catabolism is found in compound **1** treated *M. smegmatis* as more degradation metabolites including xanthine, xanthosine monophosphate and adenine are all present when compared to controls. These findings are in agreement with previous reported studies which demonstrated, at the transcriptome level, a downregulation of nucleoside biosynthesis, and an upregulation of nucleotide catabolism in nitrogen-starving mycobacteria (Jeßberger et al., 2013; Petridis et al., 2015). In addition, as folate-dependent one-carbon metabolism is essential for nucleotide synthesis, our results (Figure 2B and Supplementary Figure S2B) fit well to a nitrogen-limited intake hypothesis. This hypothesis is also supported by the significant decreased concentration of glutamine when compared to 2-oxo-glutarate (no significant difference) in compound **1** treated samples (Figure 2B), as the ratio of glutamine/2-oxo-glutarate is a main sensor of decreased nitrogen levels in mycobacteria (Gouzy et al., 2014).

Although it is currently not clear how compound **1** causes the observed nitrogen/amino acid starvation, some mechanisms are hypothetically possible. For example, by interacting with cell membranes (diterpenoids are widely reported to interact with eukaryotic and prokaryotic cell membranes) (Tsuchiya, 2015), compound **1** could interfere with ammonium, and/or amino acid transporters, thereby, decreasing nitrogen intake from external sources. In support of this hypothesis, terpenoids interfering with amino acid transport have been previously described in eukaryotic cells (Wu et al., 2007; Wang et al., 2014; Wibowo et al., 2018). Whether this can also occur in prokaryotes is currently unknown, but our metabolomic data suggests it is a possibility. Alternatively, compound **1** may alter one of the internal enzymatic systems responsible for nitrogen utilization in mycobacteria (Gouzy et al., 2014; Petridis et al., 2015) and, thus, interferes with amino acids synthesis. This could indirectly cause the overconsumption of available amino acids and nitrogen-based compounds by alteration of different pathways. The consequences of nitrogen-limited conditions would negatively affect protein synthesis (in a different way from kanamycin and streptomycin, which bind the 30 s subunit of prokaryotic ribosomes) (Poulikakos and Falagas, 2013) as well as both DNA and RNA synthesis ultimately leading to bacteria death. However, further analyses at the molecular level are necessary to give new indications about the mechanism of action and to eventually confirm the proposed hypotheses.

In conclusion, a selective anti-mycobacterial core that may act through direct or indirect alteration of nitrogen/amino acids metabolism of the mycobacteria was identified. Further structural modifications of this core could lead to an optimization of its potency and to the development of new anti-mycobacterial molecules.

## Data Availability

All datasets generated for this study are included in the manuscript and/or the Supplementary Files.

## Author Contributions

AC and KH conceived and designed the experiments. AC conducted the experiments (compound synthesis and characterization). AC and SB contributed to the bacterial screening. AC, RB, MB, and LM contributed to the bacterial screening (metabolomics). AC prepared the original draft of the manuscript. All authors edited and revised the manuscript.

## Conflict of Interest Statement

The authors declare that the research was conducted in the absence of any commercial or financial relationships that could be construed as a potential conflict of interest.

## Supplementary Material

FIGURE S1 *Mycobacterium smegmatis* metabolic networks after reference antibiotics treatments. Amino acid and nitrogen metabolisms after treatment with **(A)** ethambutol, **(B)** isoniazid, **(C)** kanamycin and **(D)** streptomycin. All the significantly more abundant metabolites are coloured in red, while the less abundant metabolites in green (when compared to the control *M. smegmatis* samples). Identified hits with no significant changes are in orange. Corresponding names for each metabolite and pathway are also annotated.

FIGURE S2 *Mycobacterium smegmatis* metabolic networks affected by compound **1**. Compound **1** significantly affects amino acid and nitrogen (see text, Figure 2A) and, in addition, **(A)** pyrimidine and purine and **(B)** folate-dependent one-carbon metabolisms. More abundant metabolites are coloured in red, while the less abundant metabolites are coloured in green (when compared to the control *M. smegmatis* samples). Identified hits with no significant changes are in orange. Corresponding names for each metabolite and pathway are also annotated.

TABLE S1 Identification (as KEGG code) of metabolites found by positive ionization mode after compound **1** treatment.

TABLE S2 Identification (as KEGG code) of metabolites found by negative ionization mode after compound **1** treatment.

TABLE S3 Ranked enriched pathways with total number of hits found by positive ionization mode after treatment with compound **1**, significant hits (*p* < 0.001), their EASE (Expression Analysis Systematic Explorer) score (Hosack et al., 2003) their raw *p*-values (Fisher’s Exact Test, FET), and the *p*-values using a Gamma distribution (all scores calculated based on the hits at *p* < 0.001).

TABLE S4 Ranked enriched pathways with total number of hits by negative ionization mode after treatment with compound **1**, significant hits (*p* < 0.001), their EASE (Expression Analysis Systematic Explorer) score (Hosack et al., 2003) their raw *p*-values (Fisher’s Exact Test, FET), and the *p*-values using a Gamma distribution (all scores calculated based on the hits at *p* < 0.001).
