# Supplementary material for: The Anti-mycobacterial Activity of a Diterpenoid-Like Molecule Operates Through Nitrogen and Amino Acid Starvation
Source: Front Microbiol. 2019 Jun 25;10:1444. doi: 10.3389/fmicb.2019.01444 (PMC6603307; doi:10.3389/fmicb.2019.01444)
Supplement: FIGURE S1 — Mycobacterium smegmatis metabolic networks after reference antibiotics treatments. Amino acid and nitrogen metabolisms after treatment with (A) ethambutol, (B) isoniazid, (C) kanamycin and (D) streptomycin. All the significantly more abundant metabolites are coloured in red, while the less abundant metabolites in green (when compared to the control M. smegmatis samples). Identified hits with no significant changes are in orange. Corresponding names for each metabolite and pathway are also annotated. [file Data_Sheet_1.pdf]

A

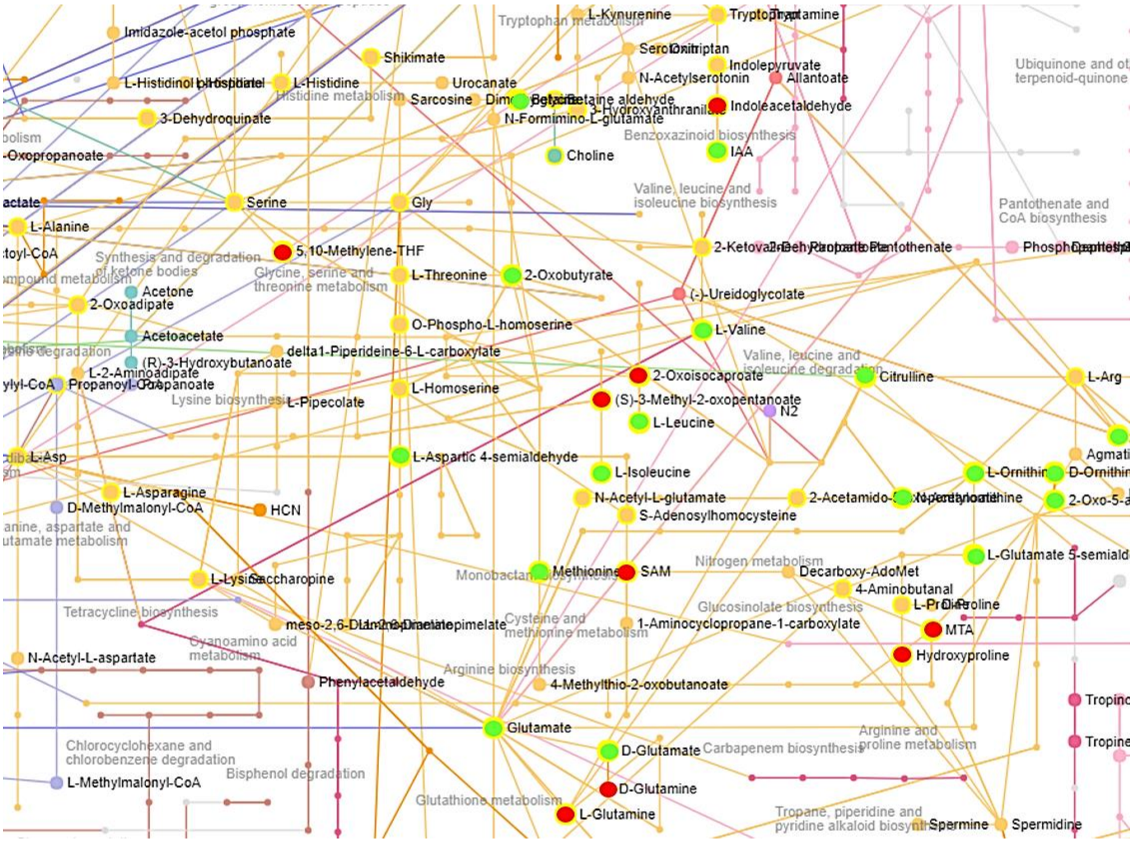

Imidazole-acetol phosphate
Tryptophan metabolism
L-Kynurenine
Shikimate
Serotonin
Indolepyruvate
N-Acetylserotonin
Allantoate
Ubiquinone and other terpenoid-quinone
L-Histidine
Histidine metabolism
Urocanate
Sarcosine
Betaine aldehyde
3-Hydroxyanthranilate
Indoleacetaldehyde
N-Formimino-L-glutamate
3-Dehydroquinate
Benzoxazinoid biosynthesis
IAA
Choline
Valine, leucine and isoleucine biosynthesis
Serine
Gly
Pantothenate and CoA biosynthesis
L-Alanine
5,10-Methylene-THF
Synthesis and degradation of ketone bodies
Glycine, serine and threonine metabolism
L-Threonine
2-Oxobutyrate
Acetone
2-Oxoadipate
(-)-Ureidoglycolate
O-Phospho-L-homoserine
L-Valine
Acetoacetate
delta1-Piperideine-6-L-carboxylate
(R)-3-Hydroxybutanoate
Valine, leucine and isoleucine degradation
L-2-Aminoadipate
2-Oxoisocaproate
Citrulline
L-Arg
Propanoyl-CoA
Lysine biosynthesis
L-Pipecolate
L-Homoserine
(S)-3-Methyl-2-oxopentanoate
N2
L-Leucine
L-Asp
L-Aspartic 4-semialdehyde
Agmatine
L-Isoleucine
L-Ornithine
D-Ornithine
L-Asparagine
N-Acetyl-L-glutamate
D-Methylmalonyl-CoA
HCN
S-Adenosylhomocysteine
Alanine, aspartate and glutamate metabolism
Nitrogen metabolism
Decarboxy-AdoMet
L-Glutamate 5-semialdehyde
Methionine
SAM
4-Aminobutanal
Tetracycline biosynthesis
Glucosinolate biosynthesis
Cysteine and methionine metabolism
1-Aminocyclopropane-1-carboxylate
MTA
N-Acetyl-L-aspartate
Cyanoamino acid metabolism
Hydroxyproline
Arginine biosynthesis
Phenylacetaldehyde
4-Methylthio-2-oxobutanoate
Tropine
Glutamate
Chlorocyclohexane and chlorobenzene degradation
D-Glutamate
Carbapenem biosynthesis
Arginine and proline metabolism
L-Methylmalonyl-CoA
Bisphenol degradation
D-Glutamine
Glutathione metabolism
L-Glutamine
Tropane, piperidine and pyridine alkaloid biosynthesis
Spermine
Spermidine

B

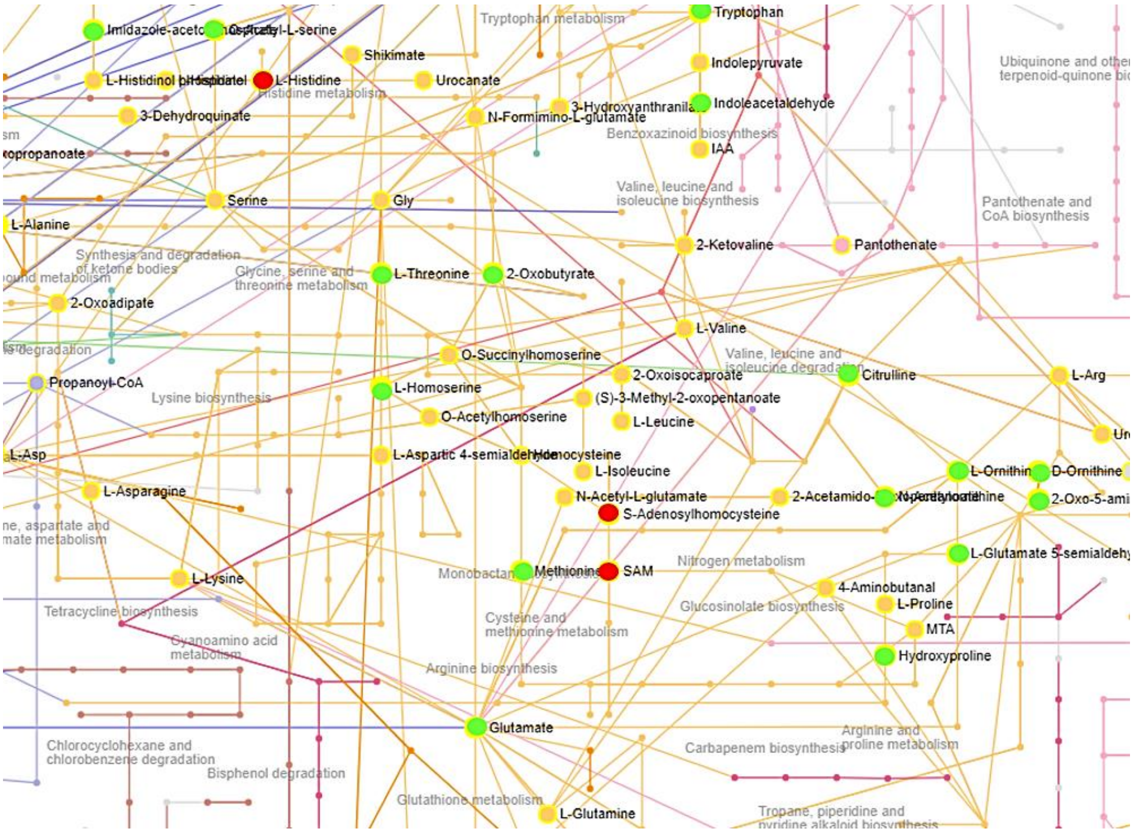

Tryptophan metabolism
Tryptophan
Shikimate
Ubiquinone and other terpenoid-quinone
L-Histidine
Histidine metabolism
Urocanate
Indolepyruvate
3-Dehydroquinate
3-Hydroxyanthranilate
Indoleacetaldehyde
N-Formimino-L-glutamate
Benzoxazinoid biosynthesis
IAA
Valine, leucine and isoleucine biosynthesis
Serine
Gly
Pantothenate and CoA biosynthesis
L-Alanine
2-Ketovaline
Pantothenate
Synthesis and degradation of ketone bodies
Glycine, serine and threonine metabolism
L-Threonine
2-Oxobutyrate
2-Oxoadipate
L-Valine
O-Succinylhomoserine
Valine, leucine and isoleucine degradation
2-Oxoisocaproate
Citrulline
L-Arg
Propanoyl-CoA
Lysine biosynthesis
L-Homoserine
(S)-3-Methyl-2-oxopentanoate
O-Acetylhomoserine
L-Leucine
L-Asp
L-Aspartic 4-semialdehyde
Homocysteine
L-Isoleucine
L-Ornithine
D-Ornithine
L-Asparagine
N-Acetyl-L-glutamate
2-Oxo-5-amino
S-Adenosylhomocysteine
L-Glutamate 5-semialdehyde
Methionine
SAM
Nitrogen metabolism
L-Lysine
4-Aminobutanal
Tetracycline biosynthesis
Glucosinolate biosynthesis
L-Proline
Cysteine and methionine metabolism
MTA
Cyanoamino acid metabolism
Hydroxyproline
Arginine biosynthesis
Glutamate
Chlorocyclohexane and chlorobenzene degradation
Arginine and proline metabolism
Carbapenem biosynthesis
Bisphenol degradation
Glutathione metabolism
L-Glutamine
Tropane, piperidine and pyridine alkaloid biosynthesis

C

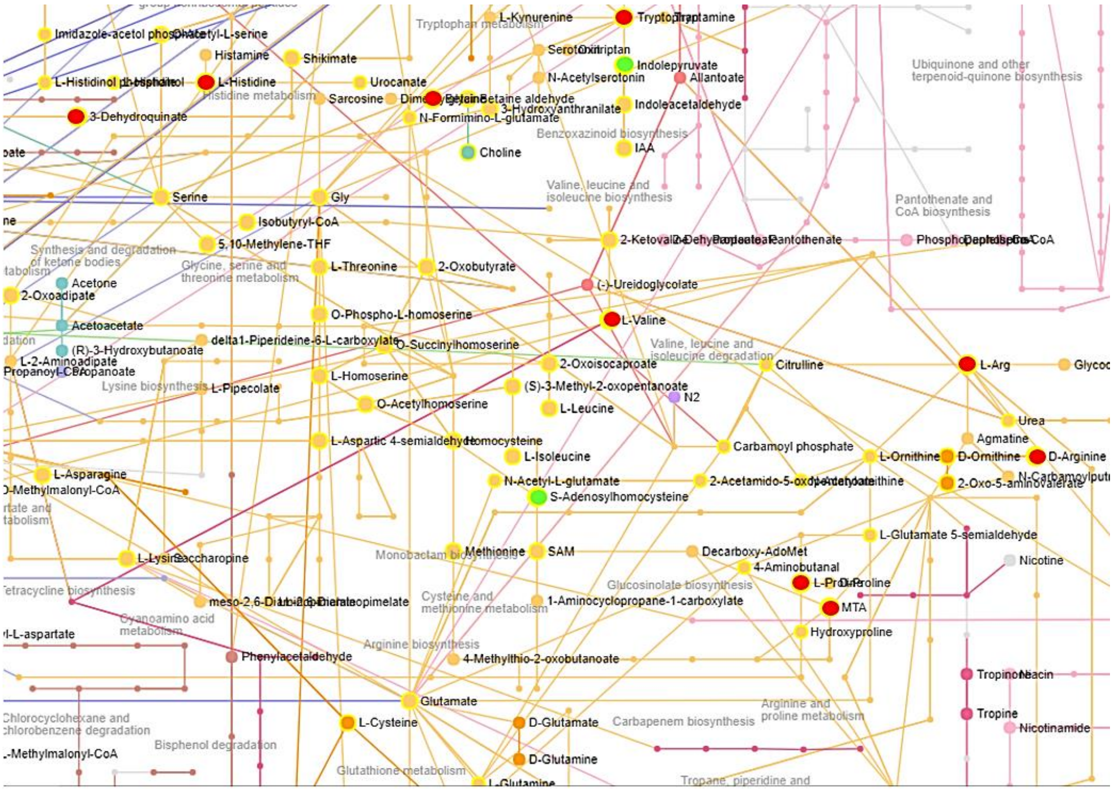

D

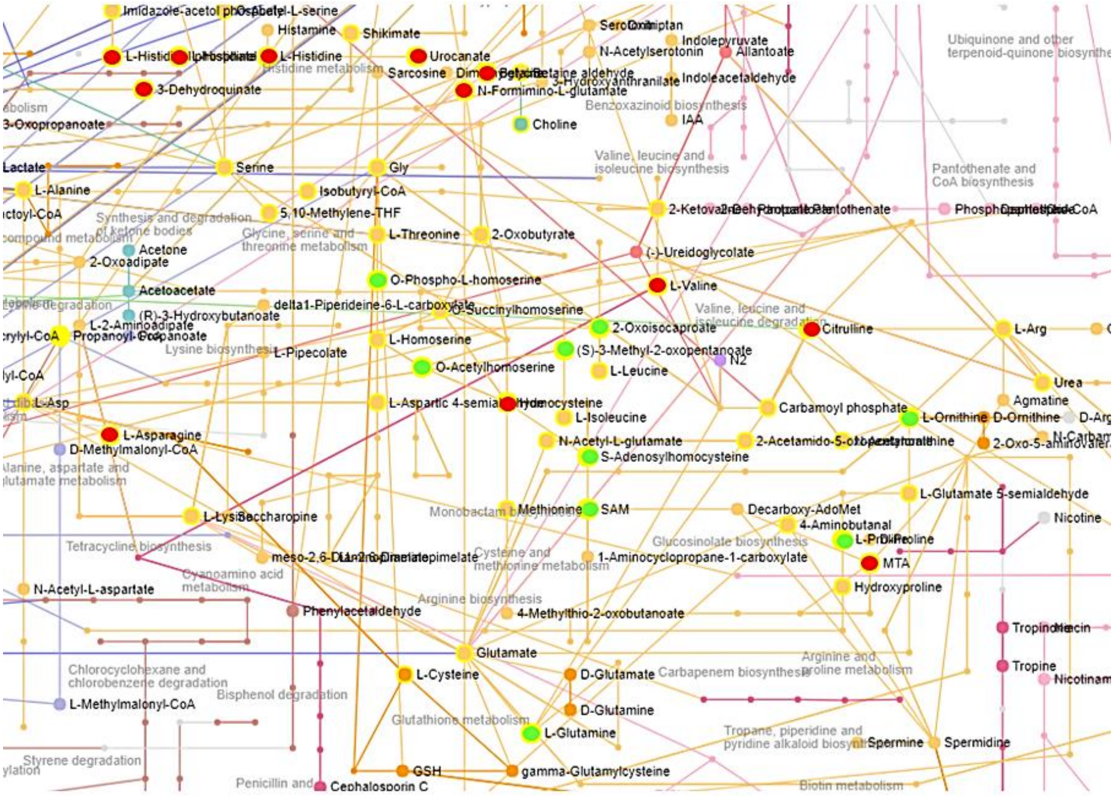

S1. *Mycobacterium smegmatis* metabolic networks after reference antibiotics treatments. Amino acid and nitrogen metabolisms after treatment with A) ethambutol, B) isoniazid, C) kanamycin and D) streptomycin. All the significantly more abundant metabolites are coloured in red, while the less abundant metabolites in green (when compared to the control *M. smegmatis* samples). Identified hits with no significant changes are in orange. Corresponding names for each metabolite and pathway are also annotated.
