# Supplementary material for: The Anti-mycobacterial Activity of a Diterpenoid-Like Molecule Operates Through Nitrogen and Amino Acid Starvation
Source: Front Microbiol. 2019 Jun 25;10:1444. doi: 10.3389/fmicb.2019.01444 (PMC6603307; doi:10.3389/fmicb.2019.01444)
Supplement: FIGURE S2 — Mycobacterium smegmatis metabolic networks affected by compound 1. Compound 1 significantly affects amino acid and nitrogen (see text, Figure 2A) and, in addition, (A) pyrimidine and purine and (B) folate-dependent one-carbon metabolisms. More abundant metabolites are coloured in red, while the less abundant metabolites are coloured in green (when compared to the control M. smegmatis samples). Identified hits with no significant changes are in orange. Corresponding names for each metabolite and pathway are also annotated. [file Data_Sheet_2.pdf]

A

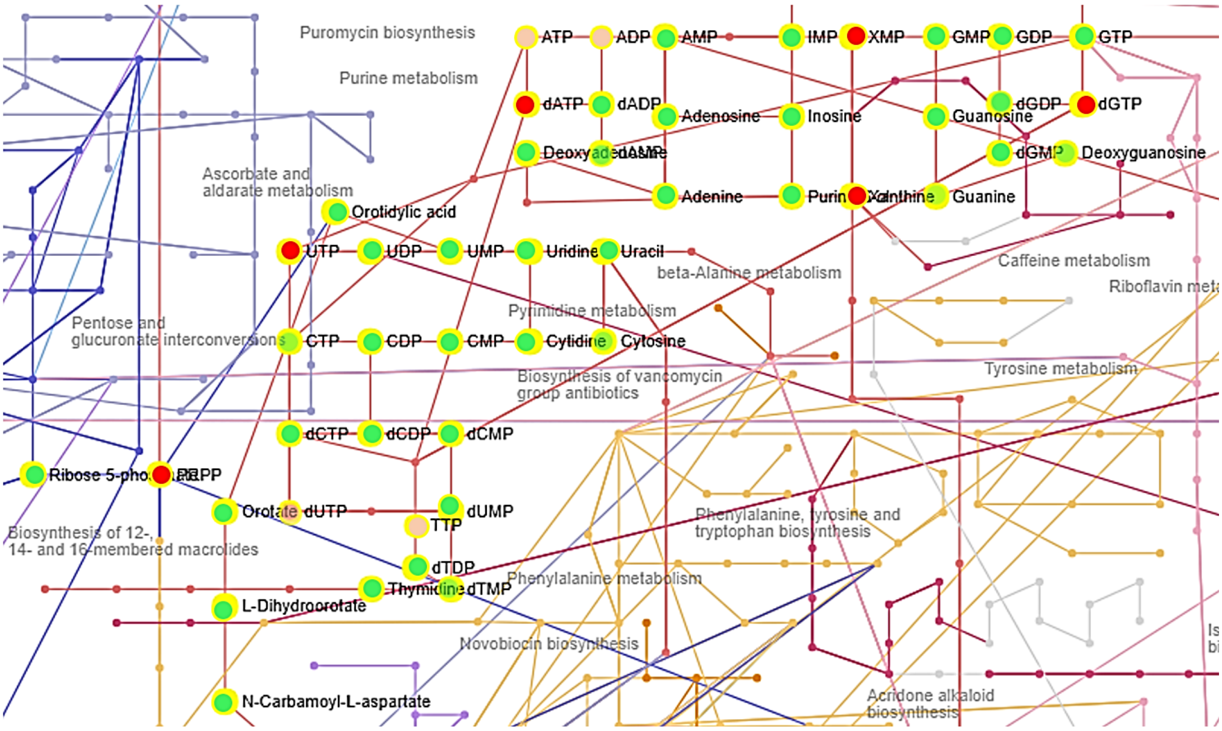

B

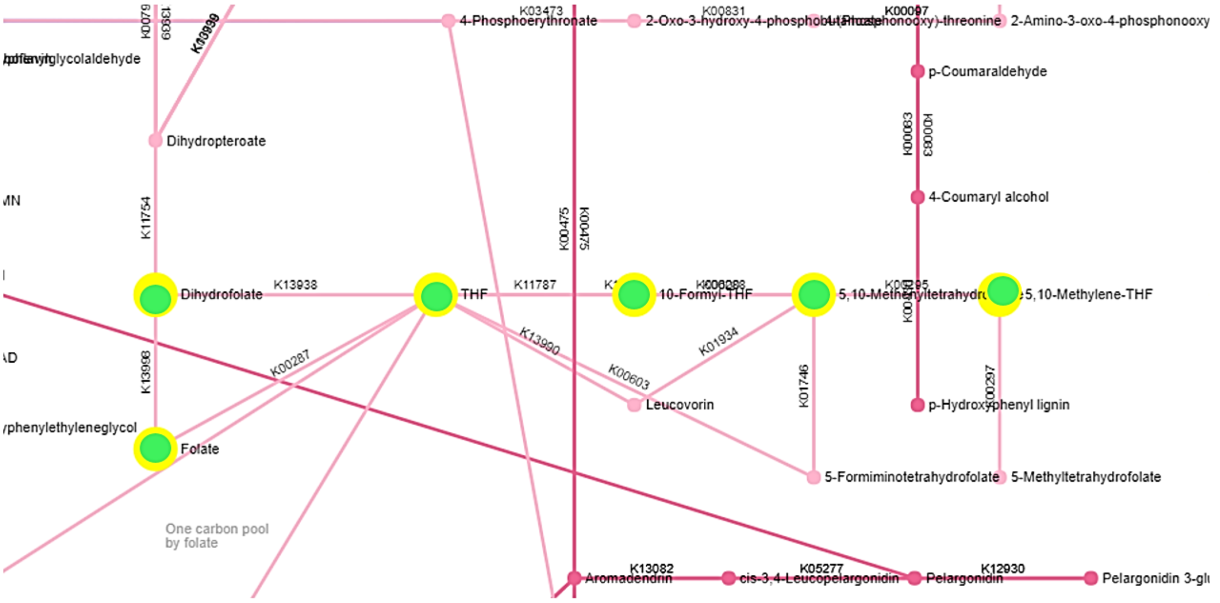

S2. *Mycobacterium smegmatis* metabolic networks affected by compound 1. Compound **1** significantly affects amino acid and nitrogen (see text, Figure 2A) and, in addition, A) pyrimidine and purine and B) folate-dependent one-carbon metabolisms. More abundant metabolites are coloured in red, while the less abundant metabolites are coloured in green (when compared to the control *M. smegmatis* samples). Identified hits with no significant changes are in orange. Corresponding names for each metabolite and pathway are also annotated.
